# A predictive model for patent ductus arteriosus seven days postpartum in preterm infants: an ultrasound-based assessment of ductus arteriosus intimal thickness within 24 h after birth

**DOI:** 10.3389/fped.2024.1388921

**Published:** 2024-04-25

**Authors:** Xin-Lu Hu, Ting-Ting Zhu, Hui Wang, Cui Hou, Jun-Cheng Ni, Zhuo-Fan Zhang, Xiao-Chen Li, Hao Peng, Hong Li, Ling Sun, Qiu-Qin Xu

**Affiliations:** ^1^ Department of Cardiology, Children’s Hospital of Soochow University, Suzhou, Jiangsu, China; ^2^ Department of Neonatology, Children’s Hospital of Soochow University, Suzhou, Jiangsu, China; ^3^ Department of Epidemiology, Suzhou Medical College of Soochow University, Suzhou, Jiangsu, China

**Keywords:** patent ductus arteriosus, intimal thickness, preterm infants, echocardiography, predictive model

## Abstract

**Objectives:**

To develop a predictive model for patent ductus arteriosus (PDA) in preterm infants at seven days postpartum. The model employs ultrasound measurements of the ductus arteriosus (DA) intimal thickness (IT) obtained within 24 h after birth.

**Methods:**

One hundred and five preterm infants with gestational ages ranging from 27.0 to 36.7 weeks admitted within 24 h following birth were prospectively enrolled. Echocardiographic assessments were performed to measure DA IT within 24 h after birth, and DA status was evaluated through echocardiography on the seventh day postpartum. Potential predictors were considered, including traditional clinical risk factors, M-mode ultrasound parameters, lumen diameter of the DA (LD), and DA flow metrics. A final prediction model was formulated through bidirectional stepwise regression analysis and subsequently subjected to internal validation. The model's discriminative ability, calibration, and clinical applicability were also assessed.

**Results:**

The final predictive model included birth weight, application of mechanical ventilation, left ventricular end-diastolic diameter (LVEDd), LD, and the logarithm of IT (logIT). The receiver operating characteristic (ROC) curve for the model, predicated on logIT, exhibited excellent discriminative power with an area under the curve (AUC) of 0.985 (95% CI: 0.966–1.000), sensitivity of 1.000, and specificity of 0.909. Moreover, the model demonstrated robust calibration and goodness-of-fit (*χ*^2^ value = 0.560, *p* > 0.05), as well as strong reproducibility (accuracy: 0.935, Kappa: 0.773), as evidenced by 10-fold cross-validation. A decision curve analysis confirmed the model's broad clinical utility.

**Conclusions:**

Our study successfully establishes a predictive model for PDA in preterm infants at seven days postpartum, leveraging the measurement of DA IT. This model enables identifying, within the first 24 h of life, infants who are likely to benefit from timely DA closure, thereby informing treatment decisions.

## Introduction

Functional closure of the ductus arteriosus (DA) usually occurs within three days after birth. Patent ductus arteriosus (PDA) has a high incidence in preterm infants, and its influencing factors, the mechanisms of which are not fully elucidated, include molecular, hemodynamic, and structural factors (1). The left-to-right shunt of a PDA in preterm infants causes adverse hemodynamic effects, resulting in complications including periventricular/intraventricular hemorrhage, pulmonary hemorrhage, bronchopulmonary dysplasia, and necrotizing enterocolitis (2).

While non-selective cyclooxygenase inhibitors, including ibuprofen and indomethacin, for PDA closure are widely demonstrated (3), pharmacological interventions do not improve outcomes compared with expectant management, and harm (including increased risk of acute kidney injury or GI bleeding) may outweigh benefit (4–6). Treatment decisions regarding prophylactic, very early, or early administration of these agents also varied considerably among centers (7). Evidence is lacking to identify which preterm infants are most likely to benefit from PDA treatment, and more effective selective treatment options for PDA remain to be elucidated (8). Some studies have investigated the relationship between serum markers, echocardiographic indicators, and hemodynamically significant PDA (hsPDA) in the neonatal period (9–11). However, few have yielded a simple predictive model for PDA in preterm infants. To date, no study has evaluated structural predictors of DA closure beyond DA diameter, length, and shape.

Recently, our group found that the intimal thickness (IT) of the DA, which can be measured by echocardiography, tended to be thinner and have a slower growth rate in newborns in whom the DA failed to close by three days of life. They also had a smaller ratio of IT to lumen diameter of the DA (LD) within 24 h after birth than that of infants with spontaneous closure (12). In this study, we describe creating a predictive model that integrates IT measurements—obtained within the first 24 h postpartum—alongside conventional echocardiographic parameters and established clinical risk factors. This comprehensive model assesses the risk of PDA at seven days postpartum. By facilitating early identification of high-risk infants, the model aids clinical decision-making and enables timely interventions to alleviate left ventricular volume load. Conversely, for infants classified as low-risk, the model allows for avoiding unnecessary pharmacologic interventions, thereby minimizing the potential for adverse side effects and reducing overall harm.

## Methods

### Study design and participants

This was a prospective cohort study focusing on preterm infants conducted from July 2020 through December 2022. A total of 105 preterm infants admitted to the Children's Hospital of Soochow University within the first 24 h following birth were enrolled in the study. Eligibility criteria included a gestational age of less than 37 weeks and a patent DA confirmed by echocardiography within the initial 24-hour postpartum window.

#### Exclusion criteria

Infants were excluded from the study for any of the following reasons:

• Presence of an atrial shunt exceeding 5 mm or other forms of congenital heart disease.
• Diagnosis of arrhythmia.
• Anatomical malformations of other systems.
• Poor quality echocardiographic images.
• Uncertain clinical outcomes owing to hospital discharge or mortality within seven days of birth.

### Clinical risk factors

The gender, gestational age (GA), birth weight, birth length, and body surface area (BSA) at birth were recorded. The presence of neonatal asphyxia, respiratory distress syndrome (RDS), or the use of mechanical ventilation within 24 h after birth were collected.

### Echocardiography examination

GE Vivid E90 and E95 (GE Vingmed Ultrasound, Horten, Norway) cardiac ultrasonic diagnostic machines were used with a 2.9–5.8 MHz phased-array probe. Echocardiographic images were collected within 24 h after birth in the supine position in a quiet state.

M-mode ultrasound data measured in real time included aortic root diameter (AO), left atrial diameter (LA), left ventricular end-diastolic dimension (LVEDd), and left ventricular ejection fraction (LVEF). The LA/AO was calculated, and body-surface-area-corrected left ventricular end-diastolic diameter (LVDb) was calculated by LVEDd/BSA.

**Figure 1:**
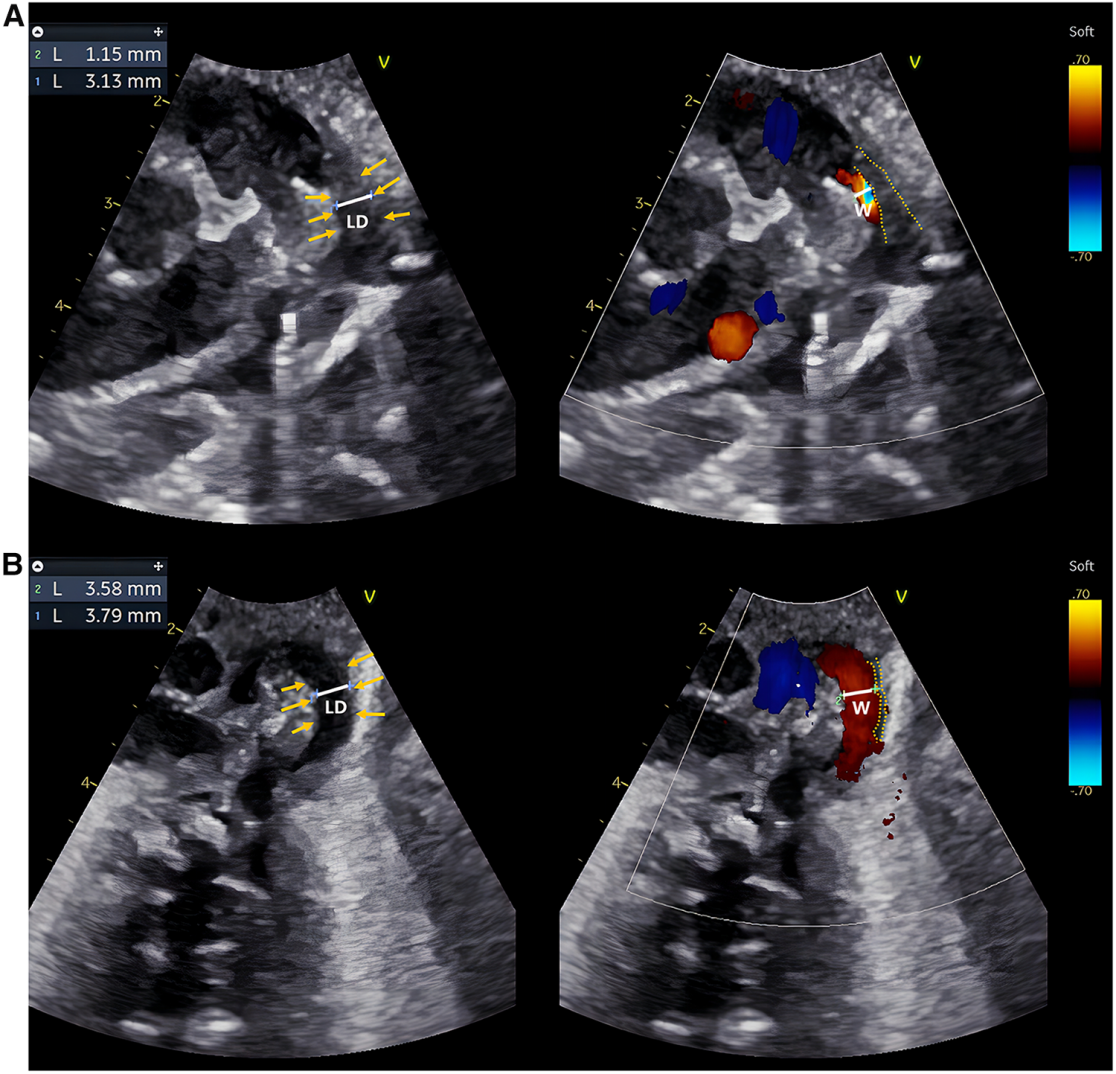
Echocardiographic IT measurements of two typical preterm infants within 24 h after birth. (**A,B**) are echocardiographic images obtained within 24 h after birth of a preterm infant in the DA-closure group and a premature infant in the DA-open group, respectively. Caliper 1: LD; Caliper 2: The width of the transcatheter flow bundle (W); Yellow arrows: inner margin of the wall of the DA; Yellow dashed line: the outline of the intima of the DA. The LD and W were measured at the same level at the narrowest point of the DA, and IT = (LD−W)/2. The intima of the DA appears hypoechoic, uneven, and loose. The IT of the preterm infant in the DA-closure group was thicker than in the DA-open group.

Dynamic images of the DA were obtained in the long-axis view at the superior sternal fossa. The direction of the shunt was recorded, the peak velocity of the left to right shunt (Vmax), and the maximum difference of the shunt velocity throughout the cardiac cycle (Vdif) were measured. (When the DA shunt is left-to-right, Vdif is the velocity difference above the spectrum baseline. When the DA shunt is bidirectional, Vdif is the absolute difference between the upper and lower baseline velocities.) The ratio of Vdif to Vmax was calculated. LD and IT were measured using an Echopac workstation off-line as described (12). Figure 1 shows the measurement of IT.

Image acquisition was performed by two trained sonographers, and measurement of DA data was performed by one experienced sonographer. All data were measured for three cardiac cycles, and the mean value was calculated. The sonographers were unaware of the clinical characteristics of the participants and the outcomes of DA closure.

Complete closure or non-closure of the DA of preterm infants by seven days after birth was confirmed by echocardiography.

## Statistical analysis

Data were analyzed using R statistical software, version 4.1.3. A two-tailed *p *< 0.05 was considered statistically significant.

## Baseline characteristics

A two-independent sample *t*-test, the Wilcoxon rank sum test, or the *χ*^2^ test was used to compare the data between the two groups. As a normal distribution was not present, the Wilcoxon rank sum test was used to compare the mean levels of IT between the two groups, and then log transformation was used to convert IT to an approximately normal distribution.

### Association of It and PDA in preterm infants seven days after birth

Univariate and multivariate logistic regression models were used to evaluate the association between IT within 24 h after birth and PDA seven days after birth in preterm infants. Given the risk factors for PDA that have been characterized, potential covariates including gender, GA, birth weight, mechanical ventilation, LA/AO, Vmax, and LD were included in the multivariate model to calculate the univariate- and multivariate-adjusted OR and 95% CI of IT on DA opening outcomes in preterm infants seven days after birth, respectively.

### Model development

Candidate predictors such as birth weight, application of mechanical ventilation, LVDb, the ratio of differential to maximum velocity (Vdif/Vmax), and LD were incorporated along with logIT into a multivariate logistic regression model. The final predictors were identified through bidirectional stepwise regression analysis. To assess multicollinearity among the predictors, VIFs were calculated. Once the final predictors were ascertained, they were employed to construct the definitive predictive model. An ANOVA was conducted to assess the improvement in fit between the initial and final models. A nomogram was subsequently created using the R package “rms” to represent the final logistic regression model graphically. This nomogram elucidates the predicted probability of PDA in preterm infants at seven days postpartum.

### Model validation

The R package “caret” was used for internal validation of the predictive model using 10-fold cross-validation, and the reproducibility of the model was tested by accuracy and Kappa value.

### Evaluation of model

The R package “riskRegression” was used to draw the receiver operating characteristic (ROC) curve, and the area under the curve (AUC) was used to evaluate the discrimination of the model. The cut-off value of the prediction probability was determined using the ROC curve. Its validity was evaluated using sensitivity, specificity, Youden index, and likelihood ratio. The positive and negative predictive values were estimated. The calibration curve was drawn, and the relationship between the observed probability and the predicted probability was used to evaluate the calibration of the model. The Hosmer-Lemeshow test was used to evaluate the goodness of fit of the model. The R package “rms” was used to calculate the Brier score to quantify the calibration of the model. The R package “rmda” was used to conduct decision curve analysis (DCA) to evaluate the clinical utility of the predictive model based on net benefit at different risk thresholds. Net reclassification improvement (NRI) was used to compare the incremental value of single predictor logIT on the prediction power of the model.

## Results

### Baseline characteristics

Initially, a total of 118 preterm infants were prospectively included, but one infant with ventricular septal defect, one with pulmonary atresia, one with pericardial effusion, two with arrhythmia, one with omphalocele, four with poor image quality, and three that were discharged from hospital within seven days after birth were excluded. Finally, 105 preterm infants were enrolled in the study, of which 56 were males and 49 were females. GA ranged from 27.0 to 36.7 weeks, with a mean GA of 33.1 weeks. The rates of spontaneous DA closure and PDA were 83.8% (88/105) and 16.2% (17/105), respectively. None of the 118 preterm infants were initially treated with ibuprofen or indomethacin or underwent surgical ligation of the DA. Eight of the 17 preterm infants in the DA-open group of enrolled infants were given oral ibuprofen suspension to promote PDA closure at 8–16 days of age (median 9 days of age). None of the 105 preterm infants underwent surgical ligation of the DA. The baseline characteristics are summarized in Table 1. Compared to the DA-closure group, the DA-open group had significantly lower GA and birth weight (*p *< 0.001). Additionally, the DA-open group exhibited a higher incidence of neonatal asphyxia, RDS, and reliance on mechanical ventilation within 24 h after birth (*p *< 0.05). Echocardiographically, the DA-open group demonstrated significantly lower Vmax) and elevated LVEDb and Vdif/Vmax ratio (*p *< 0.001). Moreover, the DA-open group had a significantly larger LD (*p *< 0.05) and a thinner IT (*p *< 0.001). Figure 1 showcases echocardiographic images from both the DA-closure and DA-open groups taken within 24 h after birth, highlighting the contrasting features of intimal thickness between the two groups.

**Table 1:** Characteristics of DA-closure group and DA-open group at seven days after birth in preterm infants.

	DA-closure group	DA-open group		
	*n* = 88	*n* = 17	*X*^2^/*t*/*w*	*p* value
Male	46 (52.3%)	10 (58.8%)	0.052	0.818
GA (weeks)	34.1 (32.3, 35.1)	30.3 (29.3, 31.1)	1,338.500	<0.001
Weight (g)	2,026 ± 480	1,392 ± 313	7.048	<0.001
Asphyxia	26 (29.5%)	11 (64.7%)	6.254	0.012
RDS	14 (15.9%)	12 (70.6%)	20.024	<0.001
Ventilation	34 (38.6%)	17 (100.0%)	19.091	<0.001
LVEF (%)	68.9 ± 5.1	68.1 ± 8.8	0.353	0.728
LVDb (mm)	117.4 (105.1, 133.1)	149.8 (141.2, 168.4)	248.000	<0.001
LA/AO	1.5 (1.4, 1.7)	1.5 (1.4, 1.7)	676.000	0.534
Vmax (m/s)	2.1 (1.6, 2.4)	1.0 (0.7, 1.4)	1,270.500	<0.001
Vdif/Vmax	0.6 (0.4, 1.3)	1.7 (1.4, 2.0)	259.000	<0.001
LD (mm)	2.4 (2.1, 3.0)	3.1 (2.5, 3.7)	416.500	0.004
IT (mm)	0.17 (0.08, 0.32)	0.03 (0.01, 0.08)	1,202.500	<0.001
LogIT	−1.8(−2.5, −1.1)	−2.5(−3.4, −1.3)	1,203.000	<0.001

GA, Gestational age at birth; RDS, respiratory distress syndrome; LVEF, left ventricular ejection fraction; LVDb, body surface area corrected left ventricular end-diastolic diameter; LA/AO, the ratio of left atrial diameter to aortic root diameter; Vmax, peak velocity of left to right shunt of DA; Vdif/Vmax, the ratio of the difference of the shunt velocity of ductus arteriosus to the peak velocity; LD, lumen diameter of DA; IT, the intimal thickness of DA; logIT, the logarithm of IT.

### Repeatability and reproducibility of IT

In our previous research, we evaluated inter-and intra-observer variability for IT measurement. The data are shown in Table 2. Our analyses showed good repeatability and reproducibility for IT measurement by echocardiography (12).

**Table 2:** Repeatability and reproducibility of intimal thickness measurement.

	Mean ± SD	Mean ± SD	Bias	95% confidence interval (bias)	*p*	95% limits of agreement
Inter-observer variability	0.16 ± 0.13	0.17 ± 0.03	−0.01	−0.05 to 0.04	0.83	−0.14 to 0.13
Intra-observer variability	0.16 ± 0.03	0.19 ± 0.03	−0.03	−0.09 to 0.03	0.26	−0.18 to 0.13

### Association of IT and PDA in preterm infants seven days after birth

Univariate analysis revealed that a smaller IT (or logIT) was significantly associated with PDA in preterm infants seven days post-partum, with an OR of 0.369 (95% CI: 0.216–0.587), (*p *< 0.001). After adjusting for GA, gender, and multiple traditional risk factors, including GA, weight, ventilation, LA/AO, Vmax, and LD, having a smaller IT (or logIT) was an independent risk factor for PDA in preterm infants at seven days post-partum, with an OR of 0.383 (95% CI: 0.188–0.691), (*p *= 0.003), and OR of 0.192 (95% CI: 0.027–0.723), (*p *= 0.004), respectively.

### Model development

Univariate logistic regression analysis revealed that weight, LVDb, Vdif/Vmax, LD, and logIT were significantly associated with PDA in preterm infants seven days after birth. Six predictors, including weight, ventilation, LVDb, Vdif/Vmax, LD, and logIT, were included in the model [there was no multicollinearity among the variables (vif > 2)]. After multivariate stepwise regression, five predictors, weight, ventilation, LVDb, LD, and logIT, were entered into the final model (Table 3). The fit of the final model was consistent with that of the initial model (*p *= 0.759). The final model-predicted probabilities were as follows:

Probability=exp(∑5i=1)/1+exp(∑5i=1)

where

∑5i=1=−28.497–0.007weight+17.459ventilation+0.047LVDb+3.163LD+0.164logIT

**Table 3:** Odds ratios from the final predictive model for PDA at seven days after birth in preterm infants.

Variables		*β*	Univariate OR (95% CI)	*p* value	*β*	Multivariate OR (95% CI)	*p* value
Weight (g)		−0.007	0.995 (0.994–0.998)	<0.001	−0.007	0.993 (0.986–0.998)	0.048
Ventilation	No						
	Yes	17.539	1.57 × 10^8^ (7.73 × 10^−33^–NA)	0.990	17.459	3.822 × 10^7^ (1.24 × 10^−128^–NA)	0.995
LVDb (mm)		0.047	1.062 (1.034–1.099)	<0.001	0.047	1.048 (1.000–1.112)	0.071
Vdif/Vmax		−0.259	4.851 (2.266–11.902)	<0.001			
LD (mm)		3.410	3.319 (1.580,7.801)	0.003	3.163	23.649 (3.886–383.885)	0.005
LogIT		−1.913	0.013 (0.002–0.057)	<0.001	−1.808	0.164 (0.026–0.553)	0.017

Ventilation, the use of mechanical ventilation within 24 h after birth; LVDb, body surface area corrected left ventricular end-diastolic diameter; Vdif/Vmax, the ratio of the difference of the shunt velocity of DA to the peak velocity; LD, lumen diameter of DA; LogIT, the logarithm of IT.

**Figure 2:**
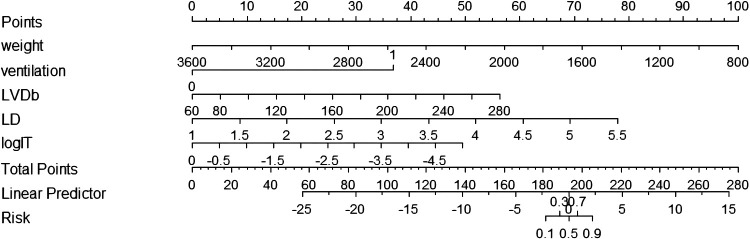
Nomogram for predicting the probability of PDA at seven days after birth in preterm infants. Ventilation, the use of mechanical ventilation within 24 h of life; LVDb, body surface area corrected left ventricular end-diastolic diameter; Vdif/Vmax, the ratio of the difference of the shunt velocity of DA to the peak velocity; LD, lumen diameter of DA; LogIT, the logarithm of IT.

To facilitate the clinical application of the predictive model, the nomogram can be used to manually obtain the prediction probability of PDA in preterm infants seven days after birth (Figure 2).

### Model validation

Using 10-fold cross-validation, we found that the accuracy of the predictive model was 0.935, and the Kappa value was 0.773, indicating that the model had good reproducibility.

### Evaluation of the model

**Figure 3:**
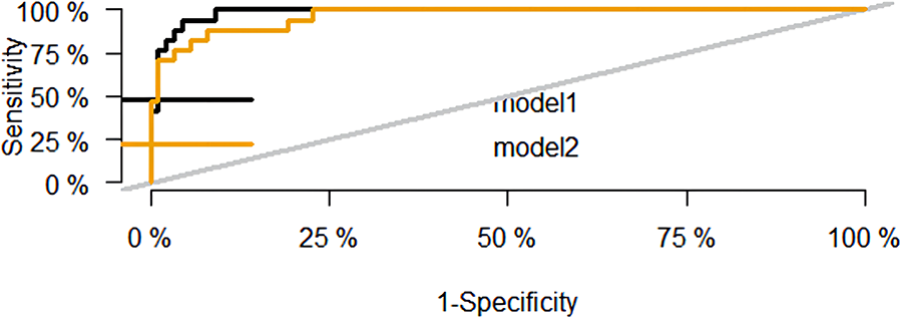
ROC curve of the predictive model for PDA at seven days after birth in preterm infants. The black curve is the ROC curve of the final predictive model based on logIT (model 1), and the yellow curve is the ROC curve of the predictive model without logIT (model 2).

Figure 3 and Table 3 show that the AUC of the final predictive model (model 1) is 0.985 (95%CI: 0.966–1.000), indicating good discrimination. Using ROC curve analysis, we found that the sensitivity, specificity, and Youden index of its diagnostic score were 100.0%, 90.9%, and 0.909, respectively, which were better than those of the predictive model without logIT (model 2) (AUC = 0.963, sensitivity = 88.2%, specificity = 92.0%, Youden index = 0.802), confirming that logIT had a significant effect on the diagnostic performance of the predictive model. In addition, both model 1 and model 2 had large positive and small negative likelihood ratios, indicating that these diagnostic tests were reliable. Table 4 also shows the positive and negative predictive values of model 1 and model 2.

**Table 4:** The evaluation of the cut-off value of the predictive model for PDA at seven days after birth in preterm infants.

Evaluation indicators	Model 1	Model 2
Cut-off value	0.114	0.251
AUC	0.985 (0.966–1.000)	0.963 (0.925–1.000)
Sensitivity	1.000	0.882
Specificity	0.909	0.920
Youden's index	0.909	0.802
Positive likelihood ratio	11.000	11.092
Negative likelihood ratio	0.000	0.128
Positive predictive value	0.680	0.682
Negative predictive value	1.000	0.976

Model 1 is the final predictive model based on logIT, and model 2 is the model with logIT removed.

**Figure 4:**
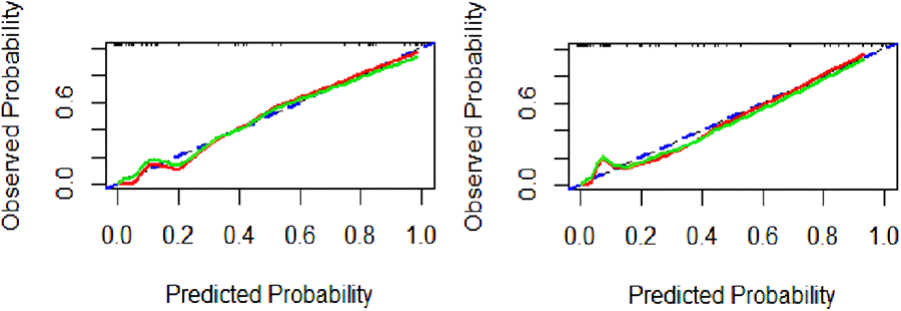
Calibration curve of the predictive model for PDA at seven days after birth in preterm infants. Model 1(**A**), model 2(**B**). The blue dashed line represents the ideal agreement between the observed and predicted probabilities. The solid red line represents the actual agreement between the observed and predicted probability. The green solid line represents the actual agreement of the correction.

Using the calibration curve (Figure 4), we found that the observed and predicted PDA probability had a high degree of overlap, indicating that the predictive model had a good calibration. Hosmer-Lemeshow test results showed that the goodness of fit of model 1 (*χ*^2^ value 0.560, *p *= 0.9998) and model 2 (*χ*^2^ value 4.418, *p *= 0.8176) were satisfactory. The Brier score of model 1 was 0.035, indicating that the final model based on logIT had a good calibration, which was better than model 2 (Brier score 0.052).

**Figure 5:**
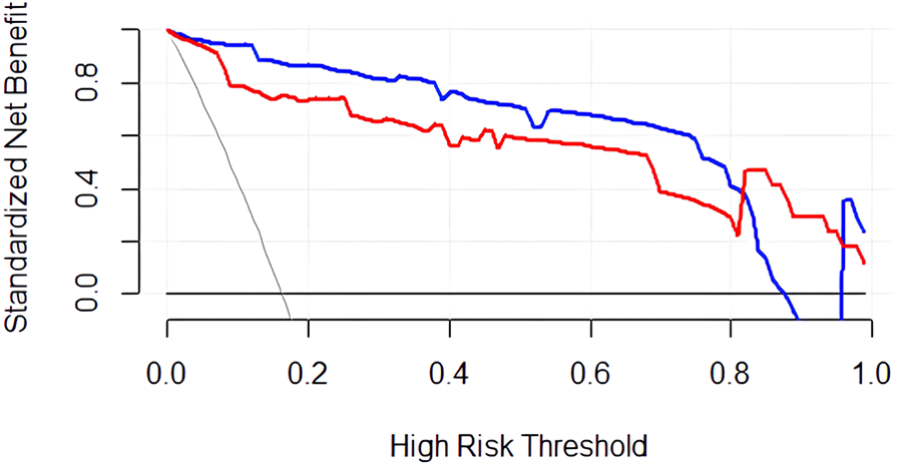
DCA curve of the predictive model for PDA at seven days after birth in preterm infants. The blue curve represents the net clinical benefit of model 1. The red curve represents the net clinical benefit of model 2.

The DCA curve (Figure 5) shows that between threshold probabilities of 0.1–0.8, the net benefit of model 1 was higher than that of model 2. The final predictive model can be applied to clinical practice with broad applicability.

In addition, the NRI was 1.374, 95% CI: 0.995–1.717 (*p *< 0.001), and the proportion of correct reclassification in model 1 was increased by 137.133% compared with model 2. The addition of logIT improved the prediction accuracy of the final predictive model.

## Discussion

Current medical treatment of PDA in preterm infants during hospitalization can be divided into preventive treatment delivered within 24 h after birth, very early treatment delivered within the first three days of life, and early treatment delivered within the first seven days of life (13, 14). However, due to the high probability of spontaneous closure of the DA in preterm infants (15), there is controversy about treatment for PDA, both in terms of which infants to treat and when to start medications. Specific treatment options for PDA vary significantly among institutions, and different physicians may make different treatment decisions (7).

A recent meta-analysis of 14 randomized controlled clinical trials comparing the outcome indicators of very early treatment, early treatment, and expectant management of hsPDA found that very early or early drug treatment of hsPDA may not reduce the mortality rate or the incidence of PDA complications in preterm infants, but very early treatment of hsPDA may reduce the length of hospital stay in preterm infants (14). By capturing predictor data within the first 24 h postpartum and correlating them with DA status at seven days postpartum, this study aims to rapidly identify infants who might benefit from early PDA closure. Such early identification enables the initiation of treatment at an exceptionally early stage or even the deployment of preventive measures. The ultimate goals are to minimize hospital stay duration and forgo interventions for preterm infants likely to experience spontaneous DA closure, as either very early or early treatments could prove deleterious.

The clinical variables incorporated into the predictive model developed in this study are readily accessible. In most healthcare settings, echocardiographic evaluations are standard procedures for preterm infants in the first 24 h after admission to neonatal intensive care units. These evaluations routinely gather data on EF, LD, and DA flow parameters, among other metrics. By dedicating just a few additional minutes, a skilled sonographer can directly measure IT on the echocardiography machine itself, obviating the need for an auxiliary workstation. This aspect enhances the model's practical utility in a clinical context. However, IT differed from the pulmonary artery end to aortic end of the DA, and thus, a slight difference in the measured IT could lead to variation. The dynamic images were played back during systole to the frame in which the flow bundle and the intima were best displayed, and then measurements on different frames of the image resulted in differences. Our analyses showed good repeatability and reproducibility, indicating that small differences in the measured level or frame had only a minor influence on the IT results.

In the multivariate logistic regression model, significant differences existed in GA, weight, RDS, ventilation, LVDb, Vmax, Vdif/Vmax, IT, and logIT between the DA-closure group and the DA-open group in the baseline data. Weight was included and GA was not included among the candidate predictors, due to the well-known strong correlation between weight and GA. Ventilation was included and RDS was not included, since ventilation is a common treatment for RDS in neonatal care units. Vdif/Vmax was included and Vmax was not included, due to the apparent collinearity of Vmax and Vdif/Vmax.

Permanent DA closure is a nuanced process involving both functional closure through muscle contraction and anatomical closure via morphological and molecular remodeling. This complex process is modulated by multiple regulatory mechanisms, including but not limited to prostaglandin E2 and arterial oxygen partial pressure (16–18). A series of histological changes ensue, including the deposition of extracellular matrix under the endothelium, disintegration of the inner elastic layer, a loss of elastic fibers in the media, and migration of smooth muscle cells into the media. The contraction of the DA lumen and intimal growth co-occur after birth, eventually forming the arterial ligament (19–21). In our previous study, we described the ultrasonic imaging characteristics of DA structure and measured IT using echocardiography, which previously had been described only by histopathology. We found that neonates without spontaneous DA closure at three days of life tended to have thinner IT and slower IT growth rates. These neonates also had a lower IT/LD within 24 h after birth than those with spontaneous DA closure by three days of life (12). In this study, the predictive model based on IT demonstrated high internal validation accuracy. The final predictive model, including logIT, increased the model's discrimination degree without IT and the diagnostic efficiency. Its clinical diagnostic results are reproducible, and it has good calibration and broad clinical applicability.

Consistent with previous studies, GA and birth weight significantly influenced spontaneous DA closure (8, 22, 23). Since birth weight is directly related to gestational age, only birth weight was included in the model construction. Here, neonatal asphyxia and RDS incidence were higher in the DA-open group. Since asphyxia or RDS is often treated with mechanical ventilation in the neonatal intensive care unit (24), previous studies have found that mechanical ventilation is independently associated with an increased risk of requiring treatment for PDA (25). Invasive respiratory support is an important predictor of requiring surgical intervention for PDA (26). We, therefore, included only the use of mechanical ventilation in the model construction. Both birth weight and mechanical ventilation were used in the final model.

Previous studies have found that abnormal DA flow patterns predict hsPDA, and parameters such as Vmax or ratio of Vmax to end-diastolic velocity can predict spontaneous DA closure (2, 11, 27). Since the shunt volume of the DA is determined by the DA resistance and the pressure gradient between the aorta and the pulmonary artery, the former depends on the intrinsic properties of the DA shape and diameter. The latter can be reflected by Vmax (28, 29). We speculated that Vdif reflects the fluctuation of the pressure gradient between the aorta and the pulmonary artery and could be used as a surrogate for the flow pattern. The larger the Vdif, the more significant the fluctuation and the more tendency toward an abnormal DA flow pattern (i.e., a growing or pulsatile pattern) (2). We introduced a new parameter in this study, Vdif/Vmax, to account for this dual effect. Vdif/Vmax differed significantly between the two groups and significantly correlated with PDA in univariate regression. However, it was omitted in the final regression model. The utility of this new DA flow parameter merits further study.

LD and LA/AO are common echocardiographic parameters used in previous studies to predict a future PDA requiring intervention (27, 30–32). In this study, LD differed significantly between the two groups and entered the final predictive model. Increased LVEDd is associated with hsPDA (1, 2), which was included in the model construction parameters after correction with BSA to account for the effect of birth weight. LVDb was also included in the final predictive model.

Several limitations must be acknowledged in the interpretation of this study's findings. First, the small sample size restricts external validation and call into question the generalizability of the predictive model. This was a single-center study in southern China, and IT was only reported by our team in the study population of our center. We look forward to future multi-center research with different races and across different regions. Furthermore, the study did not conduct subgroup analyses focusing on very premature or low birth weight infants, potentially limiting the model's specificity for high-risk populations. However, the debate over very early treatment, early treatment, and expectant management is an issue faced by all preterm infants younger than 37 weeks of gestation (14). We intend to expand the sample size in future studies with the goal of achieving a model that will be more representative of younger preterm infants in future studies.

In conclusion, this study successfully develops a predictive model for PDA in preterm infants seven days postpartum based on the ultrasound measurement of IT of the DA within 24 h after birth. This model facilitates the early identification—within 24 h postpartum—of infants that may benefit from DA closure, thereby informing treatment decisions.

## Data Availability

The raw data supporting the conclusions of this article will be made available by the authors, without undue reservation.
